# Bicalutamide 25 mg combined with minoxidil 1 mg versus minoxidil 1 mg for female pattern hair loss: A randomized double-blind clinical trial

**DOI:** 10.1016/j.jdin.2024.12.002

**Published:** 2025-01-10

**Authors:** Ricardo da Silva Libório, Adriana Viana da Motta, Hélio Amante Miot, Paulo Müller Ramos

**Affiliations:** a Departamento de Dermatologia, FMB-UNESP, Botucatu, SP, Brazil; b Hospital do Servidor Público Municipal de São Paulo, São Paulo, Brazil

**Keywords:** androgenetic alopecia, bicalutamide, female pattern hair loss, oral minoxidil, treatment, woman

## Abstract

**Background:**

Antiandrogenic drugs are often used to treat female pattern hair loss (FPHL) despite limited evidence supporting their use. There is growing interest in bicalutamide for this purpose, but its efficacy in treating FPHL has not been evaluated in clinical trials.

**Objectives:**

To assess the efficacy of 25 mg/d bicalutamide combined with 1 mg/d minoxidil compared to 1 mg/d minoxidil monotherapy over 24 weeks for FPHL treatment.

**Methods:**

A randomized, controlled, double-blind clinical trial enrolled 74 participants into 2 groups: bicalutamide 25 mg/d plus minoxidil 1 mg/d or placebo plus minoxidil 1 mg/d for 24 weeks. The primary outcome was the change in total hair density in the target area.

**Result:**

Sixty-four (86.5%) participants completed the study (32 per group). There was a mean increase of 18.1 hairs/cm^2^ in the bicalutamide-minoxidil group and 21.5 hairs/cm^2^ in the minoxidil group (*P* = .86). According to the global consensus analysis of clinical photographs, there was no difference in clinical improvement between the groups (*P* = .78).

**Limitations:**

Single-center study and short follow-up period (24 weeks).

**Conclusion:**

Bicalutamide 25 mg/d combined with minoxidil 1 mg/d did not provide additional improvement in FPHL treatment compared to minoxidil alone after 24 weeks.

**Capsule Summary**

• Bicalutamide has been shown to be effective in treating female pattern hair loss in retrospective studies. However, its effectiveness has not yet been evaluated in clinical trials.
• In this randomized clinical trial, bicalutamide associated with minoxidil was not superior to minoxidil monotherapy in the treatment of female pattern hair loss.

## Introduction

Female pattern hair loss (FPHL) is the main cause of hair loss in adult women, with an estimated prevalence of 32.3%.^1^^,^^2^ It causes a significant decrease in self-esteem and a negative impact on quality of life.^3^^,^^4^

Topical minoxidil is the only Food and Drug Administration-approved drug for FPHL.^5^ Although effective, adherence to topical treatment is a challenge.^6^^,^^7^ Low-dose oral minoxidil emerged as an alternative to topical minoxidil for treating FPHL.^8^^,^^9^

Despite the low level of evidence, drugs with antiandrogenic action (spironolactone, cyproterone, and flutamide) are frequently used in the treatment of FPHL.^10^ Recently, the benefit of the antiandrogen bicalutamide in the treatment of FPHL was described in 2 retrospective studies.^11^^,^^12^ However, the efficacy of bicalutamide in the treatment of FPHL has not yet been evaluated in prospective randomized studies.

The objective of this study is to evaluate the efficacy, safety, and tolerability of bicalutamide 25 mg daily in combination with oral minoxidil at a dose of 1 mg/d compared to oral minoxidil 1 mg/d as monotherapy, for 24 weeks, in the treatment of FPHL.

## Methods

### Patients selection

Seventy-four female patients aged between 21 and 59 years diagnosed with FPHL (Sinclair scale II-IV) consented to enroll in our research conducted at a specialized clinic in Salvador, Brazil, from August 2022 to July 2023. A board-certified dermatologist (R.S.L.) established the diagnosis of FPHL based on clinical and trichoscopic evaluation.^13^

Patients with systemic arterial hypertension, heart disease, liver disease, kidney disease, and other causes of hair loss were not eligible for the study. Patients of childbearing age were only selected when using a highly effective contraceptive method and without pregnancy plans for the 12 months following the date of inclusion in the study. Those who had undergone previous treatment for hair loss in the last 6 months were not included.

### Study design

**Fig 1:**
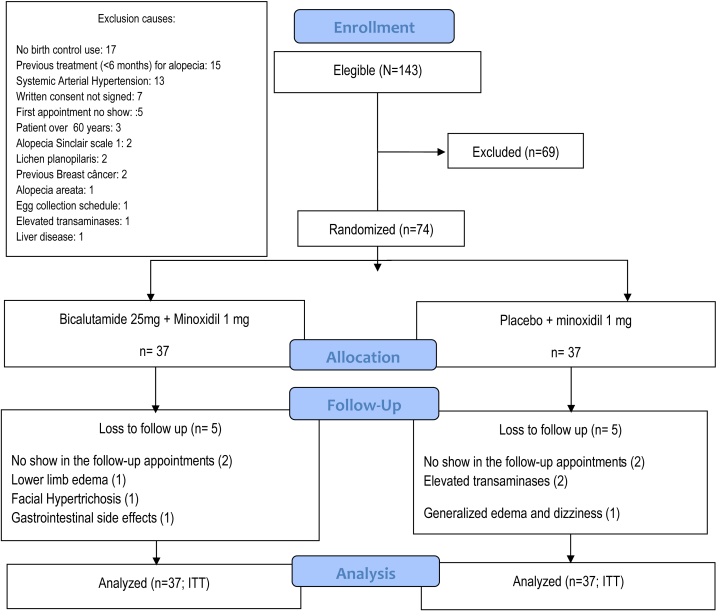
Study design. Consolidated Standards of Reporting Trials flowchart (ITT: intention-to-treat analysis).

A randomized, controlled, double-blind, parallel, and single-center clinical trial, with longitudinal follow-up for 24 weeks was performed. The participants were randomized (1:1) into 2 groups. **Group 1**: bicalutamide 25 mg and oral minoxidil 1 mg/d; and **group 2**: placebo and oral minoxidil 1 mg/d (Fig 1). The participants were sequentially allocated, and the treatment packages were distributed in opaque bottles numbered according to the computerized central randomization (in blocks) performed by a researcher not involved in the patient's evaluation.

Before starting the treatment, patients underwent manual measurement of blood pressure (BP) and heart rate, marked a visual scale of daily hair loss, and answered the Women’s Androgenetic Alopecia Quality of Life Questionnaire translated and validated to the Brazilian Portuguese.^14^^,^^15^ The patients also underwent the collection of the following blood tests: alanine aminotransferase, alkaline phosphatase, gamma-glutamyl transferase, and beta-human chorionic gonadotropin. The tests were collected again after 4 weeks, 12 weeks and 24 weeks. Elevation of liver enzymes above 2 and a half times the reference value or detection of beta-HCG would lead to the exclusion of the participan,t from the study. BP, heart rate, quality of life scale, and hair loss were also assessed at these visits.

Standardized panoramic photographs of the frontal, vertex, and parietal regions were taken with the hair parted in the middle. A circular area of 1 cm^2^ was cut, leaving hairs approximately 1 mm long in the central parietal region. This area was tattooed in 2 places, and subsequently, a trichoscopic image was performed (Dermlite DL200Hybrid dermatoscope) to count the hairs. White hairs were dyed black only in the shaved area to facilitate evaluation. These same procedures were repeated in the assessment of the 24th week of treatment. In addition, patients were questioned and examined to assess possible adverse effects (AEs).

The participants of both groups received 2 packages each. Group 1 received one package with 25-mg bicalutamide capsules to be taken once daily in the morning and 1-mg minoxidil capsules to be taken once every night. Group 2 received one package with placebo capsules to be taken once daily in the morning and 1 mg minoxidil capsules to be taken once every night. The active pharmaceuticals and placebos were prepared by a compounding pharmacy in similar packages with the same excipients. The 1 mg dose of minoxidil was chosen based on previous data regarding its efficacy and safety for FPHL.^9^^,^^16^

### Efficacy assessment

#### Primary outcome

##### Change in hair density in the target area

All hairs in the shaved target were counted in the trichoscopic image before treatment and after 24 weeks with a validated semiautomated method using ImageJ software. The standardized images were processed using a Gaussian filter (sigma = 2), followed by the wavelet operator (Mexican Hat) and estimation of the total area of hair-equivalent structures, which were divided by the area of hair-equivalent structures of 10 manually identified hairs.

#### Secondary outcomes

##### Standardized clinical photography assessment

Standardized photographs were taken and underwent consensual evaluation by 3 dermatologists blinded to the treatment. The group of dermatologists compared the initial photo with that taken 24 weeks after treatment and used a 7-point comparison scale (Global Improvement Scale): major worsening (−3), moderate worsening (−2), slight worsening (−1), no change (0), slight improvement (+1), moderate improvement (+2), and major improvement (+3).^17^

##### Assessment of hair shedding score

The score on Sinclair’s hair shedding 6-point scale was assessed at week 0 and 24.^18^

##### Assessment of quality of life

The score on the Women’s Androgenetic Alopecia Quality of Life Questionnaire was compared before the start of treatment and at the 24th week.^15^

### Statistical analysis

All patients included were analyzed by intention-to-treat and analyzed after 24 weeks of treatment; regardless of adherence to the treatment, they were randomized.^19^

Categorical variables were represented by absolute and percentage values and their 95% confidence intervals, calculated by 5000 bootstrap resamples. Their proportions were compared using Pearson χ^2^ test and χ^2^ test for trend (with exact significance).^20^^,^^21^ Continuous variables were represented by means and standard deviations or medians and quartiles (p25-p75) if indicated by the Shapiro-Wilk test. The longitudinal comparison of patient outcomes between time points was performed by mixed models, using a robust covariance matrix and probability distribution appropriate to each sample. Sequential Šidák post hoc correction was adopted. Missed data were imputed by the mixed model and Global Improvement Scale was considered as “no change.”^21^

Data analysis was performed using IBM SPSS 29.0 software (IBM). Significance was set as a (2-tailed) *P* value <.05.^22^

The sample size calculation was based on the expected increase in mean total hair density (standard deviation) of 12 (15) hairs/cm^2^ in group 2 and 24 (15) hairs/cm^2^ in group 1. It considered a power of 90%, alpha error of 5%, and dropout of up to 15%, totaling 74 participants.

## Results

Seventy-four participants with FPHL were included in the study. Table I presents the main clinical and demographic data of the sample. Most participants had mild to moderate FPHL (93.2%), and the groups were homogeneous regarding the main relevant variables (*P* > .17).

**Table I:** Main clinical-demographic characteristics of the sample, at inclusion

Variables	Group 1 (*n* = 37)	Group 2 (*n* = 37)	Total (*n* = 74)
Age (y), mean (SD)	38.7 (12.3)	35.6 (10.6)	37.2 (11.5)
BMI (m/kg^2^), mean (SD)	26.6 (5.1)	27.1 (2.7)	26.9 (5.4)
Skin phototype, *n* (%)			
I-II	13 (35.1)	13 (35.1)	26 (35.2)
III	17 (45.9)	14 (37.8)	31 (41.9)
IV-V	7 (18.9)	10 (27.0)	17 (23.0)
Menopaused, *n* (%)	10 (27.0)	4 (10.8)	14 (18.9)
Current smoking, *n* (%)	0 (−)	4 (10.8)	4 (5.4)
Heart rate (bpm), mean (SD)	75.2 (11.3)	75.2 (10.8)	75.2 (11.0)
BP (mmHg), mean (SD)			
Systolic	119.5 (13.9)	119.7 (18.8)	119.6 (16.4)
Diastolic	78.2 (7.8)	77.6 (17.0)	77.9 (13.2)
Sinclair scale, *n* (%)			
II	26 (70.3)	23 (62.2)	49 (66.2)
III	8 (21.6)	12 (32.4)	20 (27.0)
IV	3 (8.1)	2 (5.4)	5 (6.8)
WAA-QoL, mean (SD)	56.5 (22.0)	53.9 (22.6)	55.2 (22.1)
Hair shedding scale, *n* (%)			
I-II	3 (8.1)	2 (5.4)	5 (6.8)
III	2 (5.4)	4 (10.8)	6 (8.1)
IV	11 (29.7)	10 (27.0)	21 (28.4)
V	14 (37.8)	16 (43.2)	30 (40.5)
VI	7 (18.9)	5 (13.5)	12 (16.2)
Hair density (cm^2^), mean (SD)	188.2 (55.9)	188.6 (57.4)	188.4 (56.3)

Group 1: Bicalutamide + minoxidil; group 2: placebo + minoxidil.

*BMI*, Body mass index; *BP*, blood pressure; *WAA-QoL*, Women's Androgenetic Alopecia Quality of Life Questionnaire.

Of the randomized participants, 64 (86.5%) completed the 24-week study. There were 5 (13.5%) dropouts in group 1: 2 participants were unable to attend appointments, one had severe facial hypertrichosis, one had gastrointestinal AEs, and another developed severe edema in the lower limbs. In group 2, 5 (13.5%) patients did not complete the treatment: 2 were unable to attend appointments, 2 were excluded from the study after presenting transaminase elevation >2.5 times the reference value, and one did not tolerate the side effect of dizziness associated with generalized edema. The proportions of dropouts were similar between the groups.

The main outcomes are shown in Table II. The mean increase in hair density was 18.1 (95% confidence interval 3.6-32.7) hairs/cm^2^ in group 1 and 21.5 (95% confidence interval 8.1-34.2) hairs/cm^2^ in group 2. However, there was no difference in hair density variation between the groups (*P* = .86).

**Table II:** Main clinical outcomes of the 64 participants who completed the study

Variables	Group 1 (*n* = 37)		Group 2 (*n* = 37)		Difference (95% CI)	*P* value
	Baseline	Wk 24	Baseline	Wk 24		
Hair density (cm^2^), mean (SD)	188.2 (55.9)	201.1 (60.4)	188.6 (57.4)	205.3 (68.9)	−3.0 (−29.0 to 34.9)	.855
GIS, *n* (%)						
Worse	NA	2 (5)	NA	2 (5)	NA	.776
No change	NA	18 (49)	NA	15 (41)	NA	
Mild improved	NA	12 (32)	NA	16 (43)	NA	
Much improved	NA	5 (14)	NA	3 (8)	NA	
Very much improved	NA	0 (−)	NA	1 (3)	NA	
Hair shedding scale, *n* (%)						
I-II	3 (8)	9 (28)	2 (5)	8 (25)	NA	*.032*
III	2 (5)	16 (50)	4 (11)	8 (25)	NA	
IV	11 (30)	6 (19)	10 (27)	10 (31)	NA	
V	14 (38)	1 (3)	16 (43)	5 (16)	NA	
VI	7 (19)	0 (−)	5 (14)	1 (3)	NA	
WAA-QoL, mean (SD)	56.5 (22.0)	26.4 (21.9)	53.9 (22.6)	29.0 (22.5)	−2.7 (−13.5 to 8.1)	.628
Heart rate (bpm), mean (SD)	75.2 (11.3)	79.5 (10.9)	75.2 (10.8)	78.2 (10.2)	1.3 (−3.9 to 6.4)	.623
BP (mmHg), mean (SD)						
Systolic	119.5 (13.9)	120.3 (12.2)	119.7 (18.8)	119.4 (15.6)	0.4 (−6.5 to 7.2)	.914
Diastolic	78.2 (7.8)	76.2 (8.7)	74.0 (15.5)	77.6 (17.0)	−1.3 (−6.1 to 3.6)	.609

Group 1: Bicalutamide + minoxidil; group 2: placebo + minoxidil.

Italic values indicate *P* value <.05.

*BP*, Blood pressure; *GIS*, Global Improvement Scale; *WAA-QoL*, Women's Androgenetic Alopecia Quality of Life Questionnaire.

**Fig 2:**
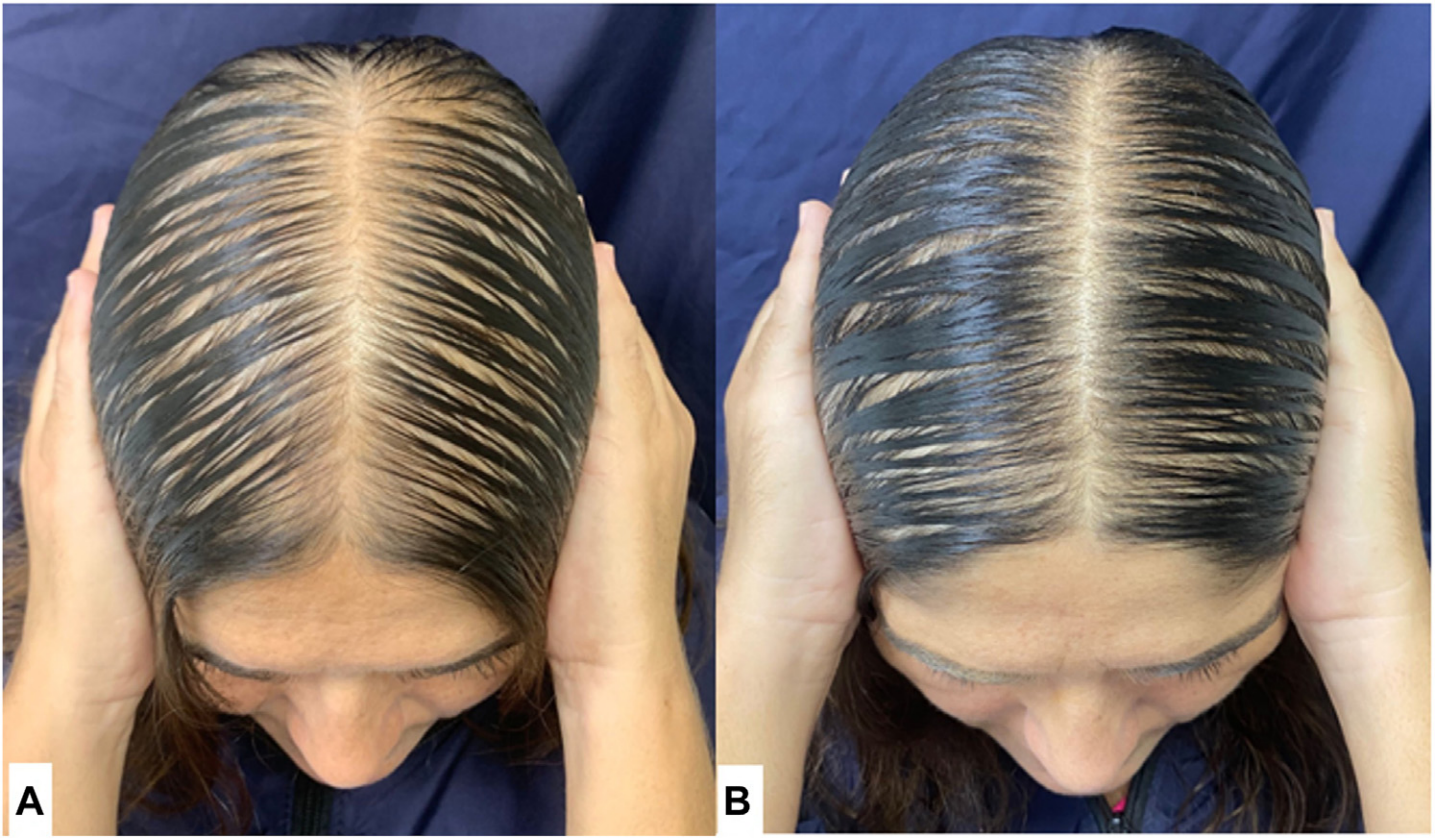
Female pattern hair loss. Improvement after 24 weeks of treatment with bicalutamide 25 mg and minoxidil 1 mg per day. **A,** Before treatment. **B,** After treatment.

**Fig 3:**
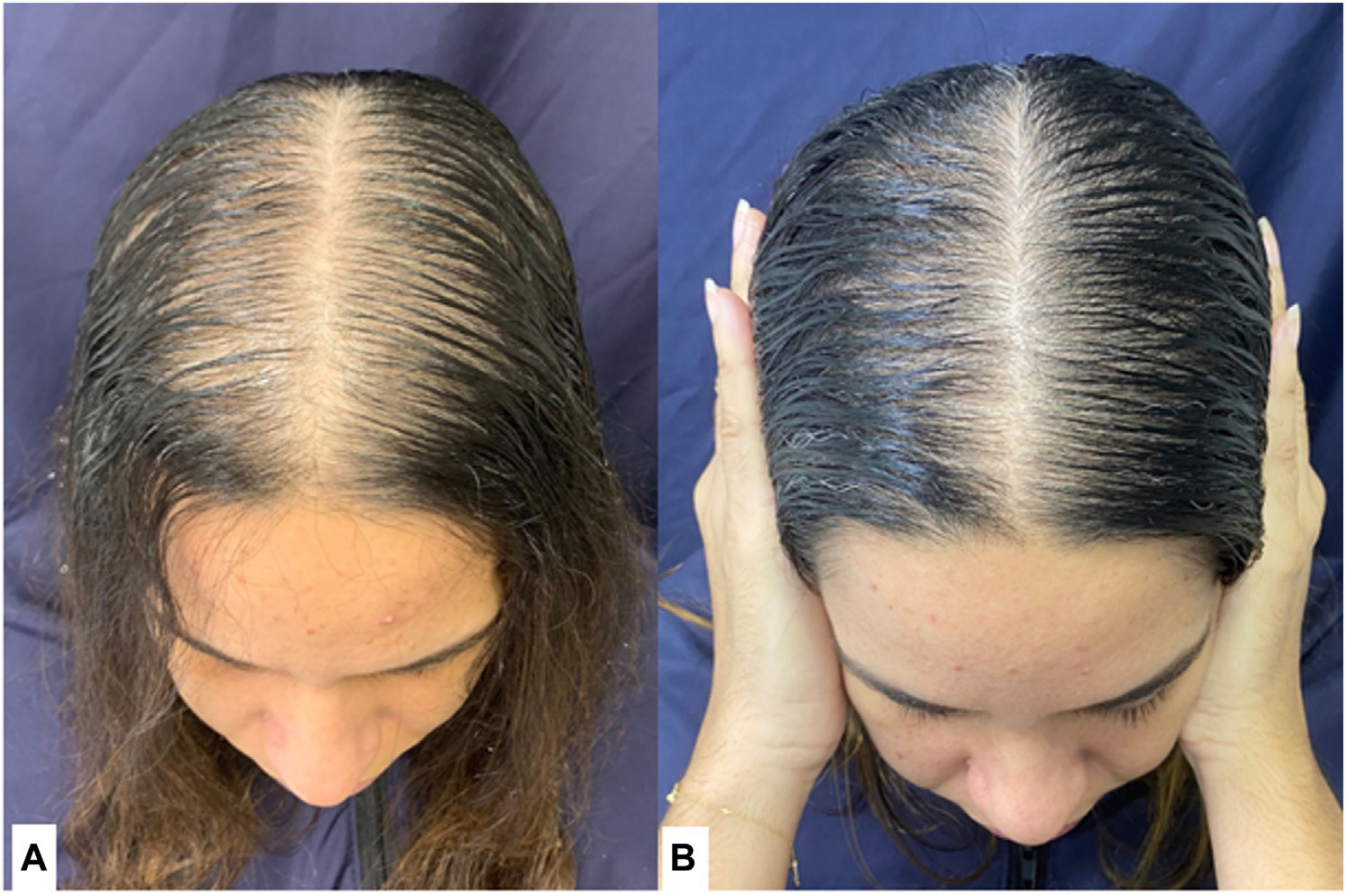
Female pattern hair loss. Improvement after 24 weeks of treatment with placebo and minoxidil 1 mg per day. **A,** Before treatment. **B,** After treatment.

According to the global consensus analysis of clinical photographs, there was no difference between the groups (*P* = .78). The group 1 presented consensus improvement assessments in 17 (46%) participants (Fig 2). In group 2, there was improvement in 20 (54%) participants (Fig 3).

Regarding Sinclair’s hair shedding scale, group 1 demonstrated a more pronounced reduction in hair shedding scores when compared to group 2 (*P* = .03). Group 1 began the study with 32 (86%) participants scoring in the highest grades of hair shedding (IV, V, and VI) and ended the study with only 7 (22%) patients. Group 2 began the study with 31 participants (84%) in the highest grades of hair shedding (IV, V, and VI) and ended the study with 16 (50%). Both groups had a marked improvement in Women’s Androgenetic Alopecia Quality of Life Questionnaire scores, with no difference between them (*P* = .63).

Three (8.1%) patients in group 1 and one (2.7%) in group 2 discontinued treatment due to AEs. The AEs presented are listed in Table III. A transient increase in hair shedding was observed during the first month of treatment in 16% of the participants in group 1 and 38% in group 2 (*P* = .04).

**Table III:** Adverse effects reported by participants during the study∗

Variables	Group 1	Group 2	*P* value	Total
First visit (4th wk)				
Gastrointestinal symptoms, *n* (%)	8 (22)	7 (19)	.778	15 (20)
Hair shedding, *n* (%)	6 (16)	14 (38)	*.036*	20 (27)
Last visit (24th wk)				
Facial hypertrichosis, *n* (%)	18 (49)	27 (73)	*.032*	45 (61)
Extrafacial hypertrichosis, *n* (%)	11 (30)	15 (41)	.465	26 (35)
Edema, *n* (%)	4 (11)	4 (11)	.999	8 (11)
Headache, *n* (%)	3 (8)	5 (14)	.711	8 (11)
Menstrual abnormality, *n* (%)	4 (11)	4 (11)	.999	8 (11)
Decreased libido, *n* (%)	3 (8)	1 (3)	.615	4 (5)

Group 1: Bicalutamide + minoxidil; group 2: placebo + minoxidil.

Italic values indicate *P* value <.05.

∗ Adverse events recorded during visits at weeks 4, 12, and 24, according to the number of participants present at each visit.

Facial hypertrichosis was the most frequent AE, and it was more prevalent among the participants in group 2 (*P* = .04). Headache, edema, menstrual abnormalities, and reduced libido were the most common side effects at the end of treatment, with no difference between groups. Gastrointestinal AEs such as bloating, nausea, and reflux were reported after 4 weeks of treatment in 15 participants (20%), with no difference between groups. Other AEs reported were dry mouth, bitter taste in the mouth, insomnia, dry skin, palpitation, and dizziness. There was no change in BP levels or heart rate before and after treatment.

Two participants in group 2 showed an increase in alanine aminotransferase greater than 2 and a half times the standard limit. Both participants were discontinued from the study for safety reasons. One month after discontinuing treatment, one of the participants showed complete improvement in the tests, and the other presented worsening. She was referred for consultation with a specialist who diagnosed bile duct disease. In group 1, there was no elevation of liver enzymes.

## Discussion

After 24 weeks, bicalutamide at a dose of 25 mg per day associated with minoxidil 1 mg/d was not superior to oral minoxidil 1 mg/d in the treatment of FPHL.

Bicalutamide is an androgen receptor antagonist used as an adjuvant in the treatment of prostate cancer. It was proposed as an alternative to flutamide because it presents high antiandrogenic potency and a better safety profile.^23^^,^^24^ Despite the possible beneficial effect of flutamide in the treatment of FPHL, it was abandoned due to the risk of severe hepatotoxicity.^25^, ^26^, ^27^

The efficacy of bicalutamide for FPHL has been evaluated in retrospective studies. The largest included 316 patients who had used this medication in doses ranging from 10 mg to 50 mg per day (mean 13.1 mg).^11^ There was an improvement in the Sinclair clinical classification of 6.5% in 3 months, 17% in 6 months, and 25% in 1 year. The second study evaluated 44 patients who received between 25 and 50 mg/d of bicalutamide (mean 41.4 mg). There was an improvement of 28% in the Sinclair clinical scale in patients who completed more than 6 months of follow-up.^12^ The concomitant use of other medications for FPHL limits the interpretation of the results.

In a 24-week retrospective comparison study of 110 Indian patients with FPHL who received spironolactone 100 mg/d or bicalutamide 50 mg/d, the mean increase in hair count was 3.2 hairs/cm^2^ in the spironolactone group compared to 5.4 hairs/cm^2^ in the bicalutamide group (*P* < .01). Photographic improvement was observed in 67.3% of patients in the spironolactone group and 84.5% of those in the bicalutamide group (*P* = .03). The mean reduction in hair shedding on the Sinclair hair shedding scale was greater in the bicalutamide group, 69.6%, compared to 51.0% in the spironolactone group (*P* < .01).^28^ In our study, the bicalutamide-minoxidil group also showed a greater reduction in the hair shedding scale scores than the placebo-minoxidil group (*P* = .04).

Although retrospective investigations demonstrated clinical improvement in FPHL with the use of bicalutamide after 6 months of treatment, this benefit was not evidenced in this randomized clinical trial. However, in the study by Ismail et al, the most significant reductions in the Sinclair scale were demonstrated in participants who used bicalutamide for 12 months.^11^ Similarly, flutamide showed progressive improvement in FPHL during the first 2 years of treatment.^25^ A more extended follow-up period could demonstrate some benefit with the use of bicalutamide.

In previous studies, Ismail et al found a mild increase in transaminases in 3% of participants (most with doses between 10 and 25 mg/d), while Fernandez-Nieto et al detected a mild increase in transaminases in 11% of participants (most with a dose of 50 mg/d).^11^^,^^12^ In the present study, there were no cases of increased transaminases in the group that used bicalutamide. Despite this, it is important to highlight the description of cases of severe hepatoxicity associated with this medication in higher doses, used to treat prostate cancer.^29^^,^^30^ Despite the rarity of such AE, dermatologists should exercise caution when prescribing this medication and managing its dosage. In addition, laboratory evaluation is suggested before starting treatment and after 4, 12, and 24 weeks.^31^

Bicalutamide showed a good tolerance profile, with no difference in the frequency of AEs between the groups. The data on adverse events in the present study are consistent with a recent retrospective study that evaluated the main AEs of oral minoxidil monotherapy, corroborating the good tolerance of bicalutamide in the sample studied.^32^

Minoxidil may cause transient hair shedding in the first 2 months of treatment due to the early release of hairs in the telogen phase (premature teloptosis). In this study, this finding was observed by 40% of participants in the placebo-minoxidil group. This frequency was similar to that previously reported by Sanabria et al in a retrospective study that evaluated 215 women with FHLP who used low-dose oral minoxidil.^32^ Interestingly, the bicalutamide group showed a lower incidence of transient hair shedding (16%; *P* = .04), suggesting a protective effect of bicalutamide against this AE.

Regarding the main AE attributed to oral minoxidil, bicalutamide was protective against facial hypertrichosis in the sample studied (49% in group 1 vs 73% in group 2; *P* = .03). This data corroborate a retrospective study that observed that the combination of oral minoxidil and bicalutamide (10-25 mg/d) reduced the incidence of hypertrichosis, allowing higher doses of oral minoxidil.^33^ These data suggest that there may be hormonal involvement in the increase in hair induced by minoxidil.

This is the first randomized clinical trial to evaluate the efficacy and safety of bicalutamide in the treatment of FPHL. Among its limitations are that it was monocentric and the limited follow-up time of the participants.

Antiandrogen receptor blockers (spironolactone, cyproterone, and bicalutamide) and 5-alpha reductase inhibitors are commonly off-label drugs used in the treatment of FPHL. However, there is a low level of evidence to support this type of treatment.^5^ Moreover, the role of the androgen factor in the physiopathology of FPHL in normoandrogenic women is still uncertain.^1^ Further clinical trials with other antiandrogens, different doses of bicalutamide, and longer follow-ups are necessary to establish the potential benefit of this kind of treatment.

In conclusion, bicalutamide at a dose of 25 mg/d associated with oral minoxidil 1 mg did not promote additional improvement in the treatment of FPHL compared to oral minoxidil 1 mg/d after 24 weeks. Longer-term clinical studies will be necessary to establish the role of bicalutamide in FPHL.

## Conflicts of interest

None disclosed.

## References

[1] Ramos P.M., Miot H.A. (2015). Female pattern hair loss: a clinical and pathophysiological review. An Bras Dermatol.

[2] Tsutsui G.M., Ramos P.M., Miot H.A. (2022). Prevalence of female pattern hair loss in a multiracial population. J Am Acad Dermatol.

[3] Hadshiew I.M., Foitzik K., Arck P.C. (2004). Burden of hair loss: stress and the underestimated psychosocial impact of telogen effluvium and androgenetic alopecia. J Invest Dermatol.

[4] Schmitt J.V., Ribeiro C.F., Souza F.H. (2012). Hair loss perception and symptoms of depression in female outpatients attending a general dermatology clinic. An Bras Dermatol.

[5] Van Zuuren E.J., Fedorowicz Z. (2017). Interventions for female pattern hair loss. JAMA Dermatol.

[6] Rossi A., Cantisani C., Melis L. (2016). Minoxidil use in dermatology, side effects and recent patents. Recent Pat Inflamm Allergy Drug Discov.

[7] Kanti V., Hillmann K., Kottner J. (2016). Effect of minoxidil topical foam on frontotemporal and vertex androgenetic alopecia in men: a 104-week open-label clinical trial. J Eur Acad Dermatol Venereol.

[8] Sinclair R.D. (2018). Female pattern hair loss: a pilot study investigating combination therapy with low-dose oral minoxidil and spironolactone. Int J Dermatol.

[9] Ramos P.M., Sinclair R.D., Kasprzak M., Miot H.A. (2020). Minoxidil 1 mg oral versus minoxidil 5% topical solution for the treatment of female-pattern hair loss: a randomized clinical trial. J Am Acad Dermatol.

[10] Ramos P.M., Melo D.F., Radwanski H. (2023). Female-pattern hair loss: therapeutic update. An Bras Dermatol.

[11] Ismail F.F., Meah N., Carvalho L.T. (2020). Safety of oral bicalutamide in female pattern hair loss: a retrospective review of 316 patients. J Am Acad Dermatol.

[12] Fernandez-Nieto D., Saceda-Corralo D., Jimenez-Cauhe J. (2020). Bicalutamide: a potential new oral antiandrogenic drug for female pattern hair loss. J Am Acad Dermatol.

[13] Gan D.C., Sinclair R.D. (2005). Prevalence of male and female pattern hair loss in Maryborough. J Invest Dermatol Symp Proc.

[14] Dolte K.S., Girman C.J., Hartmaier S. (2000). Development of a health-related quality of life questionnaire for women with androgenic alopecia. Clin Exp Dermatol.

[15] Shimizu G.K.M., Wedy G.F., Schaefer L.V. (2018). Translation into Portuguese language (Brazil), transcultural adaptation and validation of the quality of life questionnaire in female pattern hair loss (WAA-Qol-BP). An Bras Dermatol.

[16] Nascimento E Silva M., Ramos P.M., Silva M.R., Nascimento E Silva R., Barbosa Raposo N.R. (2022). Randomized clinical trial of low-dose oral minoxidil for the treatment of female pattern hair loss: 0.25 mg versus 1 mg. J Am Acad Dermatol.

[17] Narins R.S., Brandt F., Leyden J. (2003). A randomized, double-blind, multicenter comparison of the efficacy and tolerability of Restylane versus Zyplast for the correction of nasolabial folds. Dermatol Surg.

[18] Sinclair R. (2015). Hair shedding in women: how much is too much?. Br J Dermatol.

[19] Miot H.A. (2019). Anomalous values and missing data in clinical and experimental studies. J Vasc Bras.

[20] Miot H.A. (2020). Analysis of ordinal data in clinical and experimental studies. J Vasc Bras.

[21] Miola A.C., Miot H.A. (2022). Comparing categorical variables in clinical and experimental studies. J Vasc Bras.

[22] Miola A.C., Miot H.A. (2021). P-value and effect-size in clinical and experimental studies. J Vasc Bras.

[23] Ricci F., Buzzatti G., Rubagotti A. (2014). Safety of antiandrogen therapy for treating prostate cancer. Expet Opin Drug Saf.

[24] Furr B.J., Tucker H. (1996). The preclinical development of bicalutamide: pharmacodynamics and mechanism of action. Urology.

[25] Paradisi R., Porcu E., Fabbri R. (2011). Prospective cohort study on the effects and tolerability of flutamide in patients with female pattern hair loss. Ann Pharmacother.

[26] Lübbert C., Wiese M., Haupt R., Ruf B.R. (2004). Toxic hepatitis and liver failure under therapy with flutamide. Internist.

[27] Giorgetti R., di Muzio M., Giorgetti A. (2017). Flutamide-induced hepatotoxicity: ethical and scientifical issues. Eur Rev Med Pharmacol Sci.

[28] Jha A.K., Zeeshan M.D., Singh A. (2024). Efficacy and safety of spironolactone versus bicalutamide in female pattern hair loss: a retrospective comparative study. Australas J Dermatol.

[29] Trueb R.M., Luu N.C., Uribe N.C. (2022). Comment on: bicalutamide and the new perspectives for female hair loss treatment: what dermatologists should know. J Cosmet Dermatol.

[30] O'Briant C.L., Flaig T.W., Utz K.J. (2008). Bicalutamide-associated fulminant hepatotoxicity. Pharmacotherapy.

[31] Carvalho R.M., Santos L.D.N., Ramos P.M. (2022). Bicalutamide and the new perspectives for female pattern hair loss treatment: what dermatologists should know. J Cosmet.

[32] Sanabria B., de Nardo Vanzela T., Miot H.A. (2021). Adverse effects of low-dose oral minoxidil for androgenetic alopecia in 435 patients. J Am Acad Dermatol.

[33] Moussa A., Kazmi A., Bokhari L. (2022). Bicalutamide improves minoxidil-induced hypertrichosis in female pattern hair loss: a retrospective review of 35 patients. J Am Academ Dermatol.

